# Pseudotumoral Amyloidosis Presentation With Upper Digestive Bleeding

**DOI:** 10.7759/cureus.39094

**Published:** 2023-05-16

**Authors:** Igor Logetto Caetité Gomes, Alexandre de Sousa Carlos, Angelo So Taa Kum, Alexandre Moraes Bestetti, Eduardo Guimarães Hourneaux de Moura

**Affiliations:** 1 Gastroenterology, Faculdade de Medicina da Universidade de São Paulo, Sao Paulo, BRA

**Keywords:** case report, congo red, gastric, gastrointestinal hemorrhage, amyloidosis

## Abstract

*Amyloidosis* is a condition related to the extracellular deposition of abnormal fibrillar proteins. Gastric involvement may present as a systemic or localized form of the disease. The endoscopic presentation can vary from nodular, ulcerated, or infiltrative lesions. Clinical manifestations are nonspecific, including hyporexia, nausea, vomiting, weight loss, epigastralgia, and abdominal discomfort. Thus, amyloidosis can clinically and endoscopically mimic other diseases, such as neoplasms, syphilis, tuberculosis, and Crohn's disease, requiring a high suspicion. When it manifests with gastrointestinal bleeding, it most commonly occurs as intermittent melena. This report aims to present an unusual case of a patient with upper gastrointestinal bleeding exteriorized through melena secondary to amyloidosis with gastric involvement.

## Introduction

Upper gastrointestinal bleeding is the most indicated cause for the endoscopist at the emergency room, and studies show that mortality can reach 10% in this context [1]. The primary etiology is peptic ulcer disease; amyloidosis is uncommon but must be considered. It can mimic other lesions, such as gastric cancer, and confuse the endoscopist [2].

Amyloidosis is related to the extracellular deposition of abnormal fibrillar proteins, mainly affecting cutaneous, cardiovascular, renal, and neural organs [3]. The involvement of the gastrointestinal tract may present as a systemic or localized form of the disease. Exclusively gastric involvement is rare [4]. Endoscopic treatment by endoscopic submucosal dissection has been reported in the literature, particularly in patients with elevated and well-defined gastric lesions outside the context of bleeding [5,6].

This report aims to present an unusual case of a patient with upper gastrointestinal bleeding exteriorized through melena secondary to amyloidosis with gastric involvement.

## Case presentation

**Figure 1:**
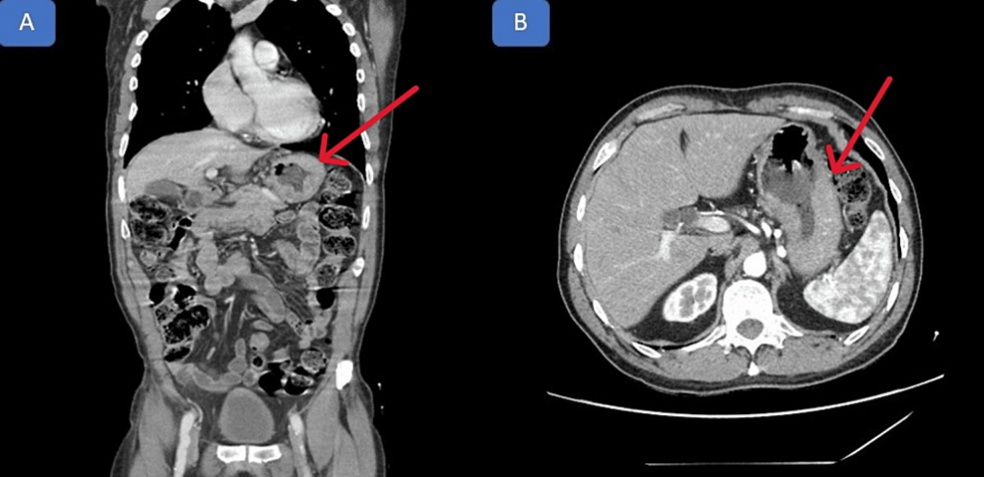
Computed tomography of the abdomen Circumferential parietal thickening of the fundus and gastric body and loss of gastric folds. Coronal plane (A) and axial plane (B).

A 65-year-old male patient who was previously healthy showed a progressive weight loss of 8 kg over six months, associated with bloating, hyporexia, weakness, and intermittent episodes of melena. Laboratory tests showed iron deficiency anemia. Computed tomography of the abdomen revealed circumferential parietal thickening of the fundus and gastric body and loss of the mucosal fold. The radiological suspicion was primary gastric neoplasia, as seen in Figure 1.

**Figure 2:**
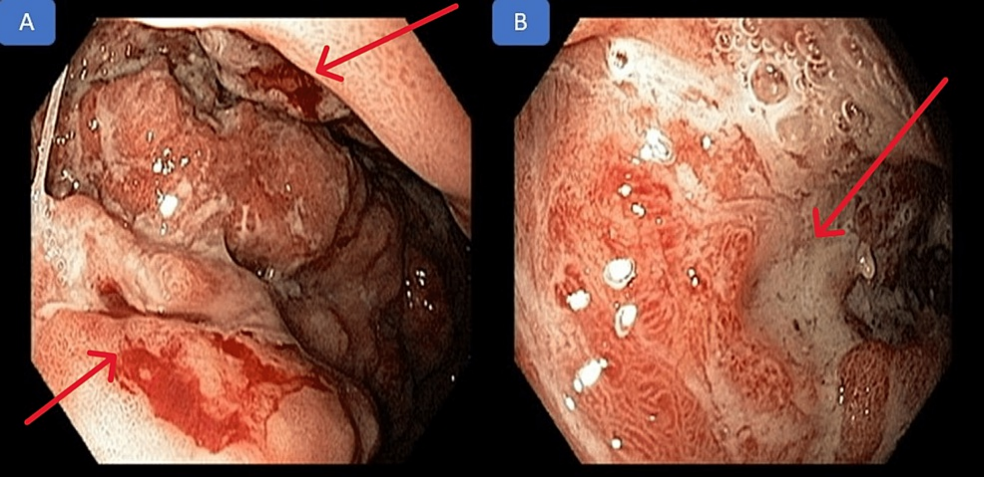
Upper digestive endoscopy An ulcerated and infiltrative lesion, measuring 5 cm, limiting organ expansion on insufflation, with self-limited bleeding (A) and covered by fibrin (B).

Upper digestive endoscopy showed the presence of an ulceroinfiltrative lesion with bleeding in self-limited, friable, partially covered by a fibrin-hematous plug, measuring about five centimeters, located two centimeters below the cardia and extending through the great curvature, anterior and posterior walls of the proximal body, limiting the expansion of the organ upon insufflation. The endoscopic appearance was compatible with the hypothesis of neoplastic lesion of the esophagogastric junction, Siewert III classification, as seen in Figure 2.

**Figure 3:**
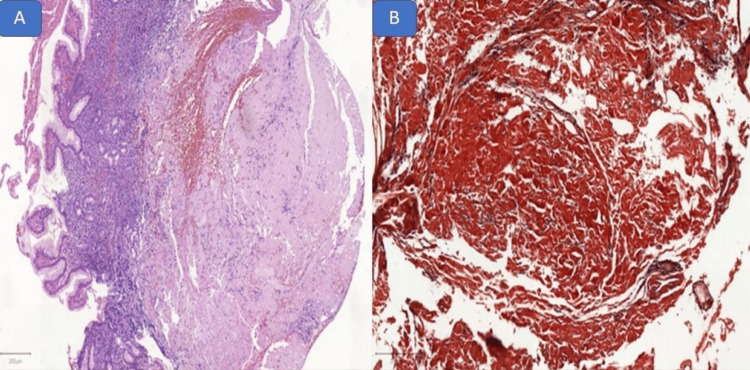
Histological analysis Microscopy showed homogeneous amorphous eosinophilic deposits in the submucosal layer (A). Congo red staining was positive for amyloid protein (B).

A biopsy of the lesion was performed. Under light microscopy, there are homogeneous eosinophilic amorphous deposits in the submucosal layer without atypia in the epithelium (Figure 3). Congo red staining was positive for amyloid protein, with green birefringence on polarized light microscopy, establishing the diagnosis of amyloidosis gastric involvement (Figure 3).

Serum protein electrophoresis revealed a monoclonal peak of the lambda chain. Bone marrow biopsy, molecular test for analysis of mutation in the transthyretin gene, skin biopsy, enterotomography, magnetic resonance imaging of the skull, computed tomography of the chest and pelvis, biopsies of the esophagus and duodenum, urinary tests, including protein research of Bence-Jones, viral serologies, rheumatoid factor, antinuclear factor, with negative results for additional alterations related to amyloidosis. Investigation with a cardiologist, nephrologist, hematologist, and neurologist failed to confirm the involvement of other organs by amyloidosis.

After the failure of clinical treatment with omeprazole, sucralfate, iron supplementation, analgesics, and support, the patient was referred for treatment with hemostatic radiotherapy, which improved the anemia and digestive bleeding.

## Discussion

Pseudotumoral amyloidosis presentation has nonspecific endoscopic findings and variable clinical manifestations. Thus, it becomes a poorly suspected condition and is rarely included among the differential diagnoses by the endoscopist. Systemic involvement may broaden the initial presentation, offering more clues to the diagnosis.

The first reported case of amyloidosis with exclusively gastrointestinal involvement occurred in 1978 [7]. In that period, the case of a 68-year-old woman with initial suspicion of gastric cancer was described. Previously, systemic amyloidosis with gastric involvement cases had already been reported [7]. Since then, less than 30 cases with this isolated involvement have been reported. In 2021, a literature scope review mentions that only 22 cases had been described up to that period [4].

From the endoscopic point of view, gastric involvement may present as nodular, ulcerated, or infiltrative lesions [8]. Additional findings include edema, enanthema, friability, erosion, retraction, and thickening of folds [9]. Clinical manifestations are nonspecific, including hyporexia, nausea, vomiting, weight loss, epigastralgia, and abdominal discomfort [10]. Thus, amyloidosis can clinically and endoscopically mimic other diseases, such as neoplasms, syphilis, tuberculosis, and Crohn's disease, requiring high suspicion [11].

When it manifests through high digestive bleeding, it is uncommon to present in massive bleeding [12]. In this context, it is more common for it to occur in the form of intermittent melena. On upper digestive endoscopy, a mucous lake with hematic content and blood clots may be found in the gastric fundus or adhered to the mucosal lesion [13].

## Conclusions

This is a rare case of pseudotumoral amyloidosis causing digestive bleeding from an ulceroinfiltrative gastric lesion. The initial suspicion was gastric cancer, and the case exemplifies one of the etiologies belonging to the group of infrequent causes of upper gastrointestinal bleeding.
